# Current state and progress of research on forensic biomechanics in China

**DOI:** 10.1080/20961790.2021.1879365

**Published:** 2021-05-04

**Authors:** Yijiu Chen

**Affiliations:** Shanghai Key Lab of Forensic Medicine, Key Lab of Forensic Science, Ministry of Justice, Shanghai Forensic Service Platform, Academy of Forensic Science, Shanghai, China

**Keywords:** Forensic sciences, forensic biomechanics, finite element analysis, multiple rigid body, postmortem human subjects (PHMS), injury reconstruction, injury criteria, material property, driver identification, anthropometric test devices, injury biomechanism

## Abstract

Forensic biomechanics gradually has become a significant component of forensic science. Forensic biomechanics is evidence-based science that applies biomechanical principles and methods to forensic practice, which has constituted one of the most potential research areas. In this review, we introduce how finite element techniques can be used to simulate forensic cases, how injury criteria and injury scales can be used to describe injury severity, and how tests of postmortem human subjects and dummy can be used to provide essential validation data. This review also describes research progress and new applications of forensic biomechanics in China.

The review shows the main research progress and new applications of forensic biomechanics in China.

The review introduces eight cases about the application of forensic biomechanics, including the multiple rigid body reconstruction, the finite element applications, study of mechanical properties, traffic crash reconstruction based on multiple techniques and analysis of morphomechanical mechanism about blood dispersal.

Though forensic biomechanics has a great advantage for the evaluation of injury mechanisms, it still has some uncertainties owing to the uniqueness of the human anatomy, the complexity of biological materials, and the uncertainty of injury-causing circumstances.

## Introduction

With the progress of the contemporary rule of law, forensic science plays an increasingly important role in legal practice and has gradually become an important means of ensuring judicial justice. Forensic scientists frequently encounter trauma related cases in practice and need to explain the causes of injury, such as the type and strength of external forces and the biomecha­nisms of injury [1]. However, traditional forensic science relies mainly upon visual observation and empirical judgment with subjective interpretation of the relationship between external forces and injuries, which is insufficient to meet modern scientific requirements for evidence—quantitative objectivity and repeatability.

Recently, biomechanics has been gradually applied in the field of forensic science. Interdisciplinary research has contributed to the rapid development of biomechanical techniques and the creation of a series of related disciplines in medicine, sports science, accident reconstruction, healthcare, and dentistry. Forensic biomechanics is evidence-based science that applies biomechanical principles and methods to forensic practice. Forensic biomechanics focuses on exploring the causation of forensic phenomena that are often deciding factors in injury, civil, and criminal cases. Forensic biomechanics has become an important branch of forensic science, providing the means for more objective and quantitative forensic investigations, and it is one of the research areas of forensic science that still have great potential for development [1–3].

This review highlights current applications of forensic biomechanics using material properties of human tissue, injury criteria, injury scales, and injury reconstruction, and describes research progress and new applications of forensic biomechanics in China.

### History and applications in medical–legal situations

Since the 1970s, the rapid development of transportation has led to an increasing number of road traffic accidents, which have fostered the integration of forensic science and biomechanics. Meanwhile, computer processing capabilities have rapidly increased allowing the use of computer technology to solve biomechanical forensic problems [4]. Marked by the publication of *Biomechanics: Mechanical Properties of Living Tissues* [5] by Fung and Skalak, forensic biomechanics formally entered the academic arena in 1982. In recent years, biomechanical analysis has been increasingly applied in forensic science to the ana­lysis of injury biomechanisms. Biomechanical analysis is commonly used to determine injury thresholds or to validate injury reconstructions, to determine injury mechanisms and cause–effect relationships; forensic biomechanical analysis has played an important role in discriminating mechanisms of injury through reconstructions [6]. Forensic biomechanics is not limited to being a science for determining cause of death but is an applied science for extrapolating and identifying mechanical causes. At present, this field has been applied to material pro­perties of human tissue, injury criteria, injury scales [7], and injury reconstruction. Biomechanical forensic evidence has been recognised and accepted by judicial systems in several countries [1, 2, 4].

### Multi-body modelling

Multi-body modelling uses rigid bodies and joints to simulate the mechanical properties of an object. The geometry of the multi-body model is usually modelled using ellipsoids and/or facets. For multi-body human body models, different contact properties and joint motion properties are usually defined to simulate the mechanical characteristics of the human body regions and joints. Similarly the geometric and mechanical characteristics of vehicle fronts can also be modelled using multi-body systems. The MADYMO pedestrian models and Chalmers pedestrian model (CPM) are the main ellipsoid multi-body human body models [8]. They were widely used with good predictions for global kinematics in vehicle-to-pedestrian collisions. But the multi-body model were limited in predicting detailed injuries because of the limitations in modelling vehicle contact characteristics used in cadaver tests. Multi-body simulation were widely used in reconstructions of real world pedestrian accidents and analysis of pedestrian dynamic response [9–12].

### Finite element (FE) modelling

FE modelling is an important tool for injury reconstruction and mechanism analysis in forensic biomechanics. FE models are extremely sensitive to parameters such as the mathematical constitutive and corresponding material properties of human tissues, and human material testing has become the basis for applying forensic biomechanics. Stress-strain curves of biological tissues are generally nonlinear, anisotropic, and viscoelastic, and non-Newtonian fluid models may better represent body fluids [13]. The material properties of human tissues have been extensively tested and applied to biomechanical modelling and case studies [13–20], and various injury criteria have been developed, such as the head injury criterion, cumulative strain damage measure, brain injury criterion, and maximum principal strain for the head [21–24] and thoracic trauma index, combined thoracic index [25], and viscous criterion [26, 27] for the thorax, providing fundamental data for biomechanical modelling and injury analysis.

### Tests on postmortem human subjects (PHMS)

The loading tests on PHMS provide data that are fundamental for biomechanical modelling and have become essential validation tools in the development of human FE models. Data from PHMS experiments conducted by Nahum et al. [28], Yoganandan et al. [29], Hardy et al. [30], and Kleiven et al. [31] have become classical validation data for head translation and rotation modelling; the head and neck impact experiments of Nightingale et al. [32] have provided validation data for neck modelling; and classical cadaveric experimental data for the trunk have been provided by frontal impact tests conducted by Kroell et al. [7], thorax belt-loading compression tests conducted by Cesari and Bouquet [33], lateral impact tests conducted by Shaw et al. [34], frontal ­abdomen impact tests conducted by Cavanaugh et al. [35], abdomen belt-loading tests conducted by Foster et al. [36] frontal pelvis impact tests conducted by Rupp et al. [37], and lateral pelvis impact tests conducted by Guillemot et al. [38]. Validation data for the limbs have mainly been obtained from experiments such as three-point bending tests, four-point bending tests, lateral loading tests, and axial loading tests [39–45]. In recent years, PHMS experiments have progressed more slowly, and new mature PHMS data are not published as frequently.

Iwamoto et al. [46] released the first version of the Total Human Model for Safety (THUMS) in 2002, a full human body FE model that includes both seated and standing models and is a faithful biological representation of the human body established by Toyota to simulate injury mechanisms in crash tests. To date, six versions of the THUMS have been released. The THUMS has been widely used for applications such as automotive safety [47], occupant protection [48], pedestrian protection [49], and accident simulation [49–52]. In 2009, Gayzik et al. [53–55] started an initiative and gradually developed the Global Human Body Models Consortium (GHBMC) model, a full human body model containing 418 parts (i.e. 179 bones described by 216 parts; 46 organs; 96 muscles; 37 blood vessels; 26 ligaments, tendons, and cartilage structures), that is still undergoing continual refinement and validation [56–59] and is gradually being applied to specific biomechanical scenarios [60, 61]. In addition, many FE models have been published for different parts of the body, such as the head, torso, and limbs [62–65]. The continuous iterative development and application of these models build upon a foundation laid in the field of forensic biomechanics.

### Specific applications of FE modelling in the literature

At present, many forensic scientists all over the world have applied the biomechanic method for simulation and analysis of real cases. Raul et al. [66] used the University Louis Pasteur of Strasbourg cranial FE model to analyse a case with two consecutive falls, and the simulation results were consistent with the actual injuries, distinguishing those obtained from the first fall and those obtained from the second fall. In the following year, Raul et al. [67] reported a case in which FE modelling was used in determining whether the deceased could have shot himself multiple times by calculating pressure and stress parameters in simu­lated brain tissue, ruling out severe damage to brain tissue from the first shot resulting in incapacitation, thereby explaining that it was possible that the deceased had shot himself multiple times. In 2007, Ejlersen et al. [68] created an FE model of the cervical spine in an attempt to explain the mechanism of cervical fracture in the deceased in an accidental death. In 2014, Kettner et al. [69] simulated a case of a baseball bat hitting the head with an FE model as well as performing physical tests, and the simulation results showing that the force of the hit could reach 17.6 kN, capable of causing serious craniocerebral injury, were consistent with the physical test results. The advantage of the FE simulation was that the simulated circumstances of the injury could be arbitrarily changed to simulate other hitting circumstances. In the same year, Matoso et al. [70] used FE simulations to compare the morpholo­gical differences of gunshot entrances between different types of bullets to help forensic experts infer which type of bullet was used. In 2017, Schenkl et al. [71] investigated a suite of methods for inferring early postmortem interval based on thermodynamic FE models of cadavers that were more precise than those based on traditional body temperature methods. In addition, forensic biomechanics has played an important role in investigating infant and child abuse cases, reconstructing traffic accidents, and analysing complex injury mechanisms [72–78], by providing innovative tools and methods to address the challenges that arise in forensic injury practice.

## Forensic biomechanics in China

Research into biomechanics started late in China. Tsinghua University, Jilin University, Hunan University, Third Military Medical University and other research institutions started to build multi-body model and FE model or to applicate business model since 2002 [10, 50, 79, 80]. The applications included vehicle safety, engineering and material mechanics, traffic injury prevention, clinical trauma research and so on. In forensic application, the Academy of Forensic Science (AFS) of the Ministry of Justice, PRC first carried out traffic accident reconstruction using numerical simulations in 2007 [81]. Reconstructions have involved automobiles, motorcycles, and bicycles, with the types of accidents including vehicle–vehicle collisions and vehicle–pedestrian collisions. AFS has completed more than 40 cases using numerical simulations and has found that forensic biomechanics can help to solve challenging problems in forensic practice, including identifying driver error, determining the pre-impact circumstances of a cyclist or pedestrian and so on. Recently, research on biological materials and blood dispersal pattern analysis has also been conducted.

### Multiple rigid body approach to address driver identification and pedestrian behaviour patterns

#### Overview

In China, the use of e-bikes and public bicycles has significantly increased because they are convenient and low cost, which has also caused traffic fatalities among cyclists to increase. Cyclists are not allowed to ride their bicycles when crossing the road or travelling against the flow of traffic; they must dismount and walk instead. Therefore, the pre-impact condition of a cyclist is an important evidence when assigning responsibility for an accident between a cyclist and the driver of a vehicle. In addition, traffic police must identify the driver responsible for each accident. Traditional approaches to accomplish this are based on vehicle traces, victim injuries, and accident traces. Recently, multi-body simulation models have been applied to reconstruct real-world road accidents. Some examples using multiple rigid body simulation in traffic accident cases are presented.

#### Case examples 1–3

**Figure 1.**
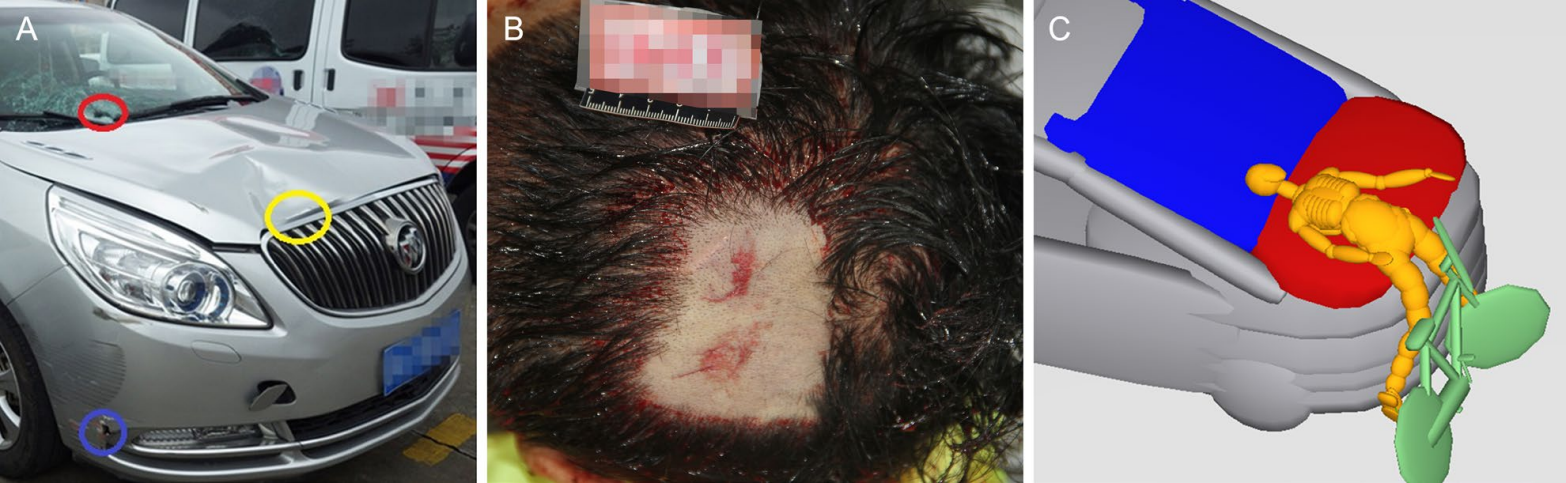
(A) Damage on the vehicle involved in the collision, (B) occipital region scalp lacerations on the cyclist, and (C) the instant of head–windshield impact in the result that showed the most likely scenario. Adapted with permission from [82].

Case 1 is a vehicle–bicycle accident and the key was to identify the pre-impact condition of a cyclist (whether the cyclist had been walking or cycling) [82]. Multi-body simulation combined with a multi-objective genetic algorithm and three-dimensional (3D) motion capture were conducted. The motion capture results were used to define the posture of the human model during walking and cycling simulations. Pre-impact parameters of the models were treated as unknown design variables, and the genetic algorithm was used to find optimal solutions. The objective result indicated that the cyclist was more likely to have been walking with the bicycle than riding on the bicycle. The optimised result showed that all observed contact points matched, and the injury parameters correlated well with the real injuries sustained by the cyclist (Figure 1).

**Figure 2.**
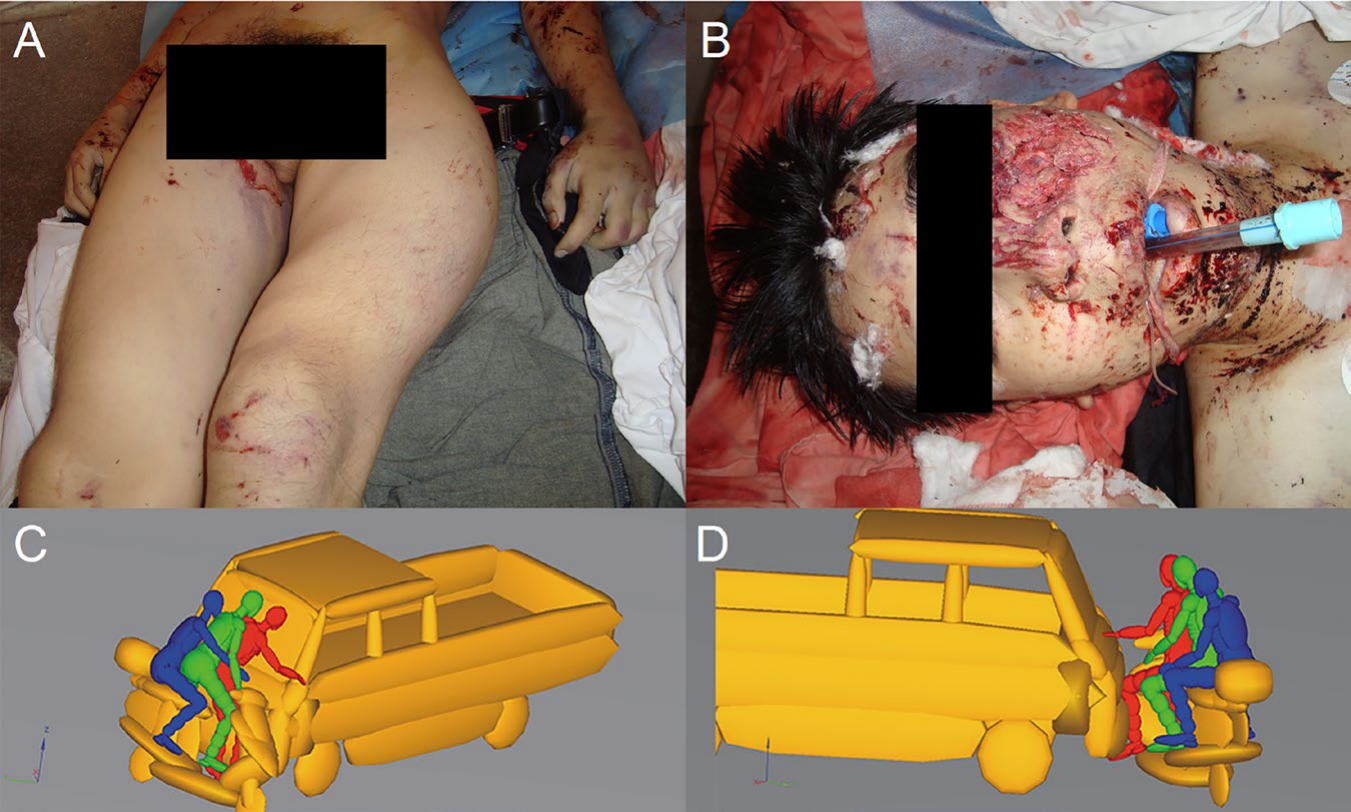
The injuries suffered by the driver: (A) left-femur fracture; (B) left-cheek skin lacerations, nasal bone and left-cheekbone fractures; (C) the instant of head injuries suffered in the simulation; (D) the instant of injuries of left leg suffered in the simulation. Adapted with permission from [83].

In Case 2 and Case 3, the aim was to identify the driver and passengers in scooter or motorcycle-related traffic accidents [83]. In Case 2, three males had ridden a scooter through an intersection. In the collision, the riders were thrown from the scooter to the ground. The injuries of the riders were limited mainly to the head and lower limbs. The MADYMO simulations yielded the kinematics of the three rider models and the scooter model as well as the injuries of the riders. The head injury criterion (HIC) was used to assess the severity of head injury and impact load force to predict the fracture. The injury results indicated that the driver suffered severe head injury, left-femur fracture, left-tibia and fibula fracture, which correlated well with one of the three riders. Therefore, the driver was identified (Figure 2).

**Figure 3.**
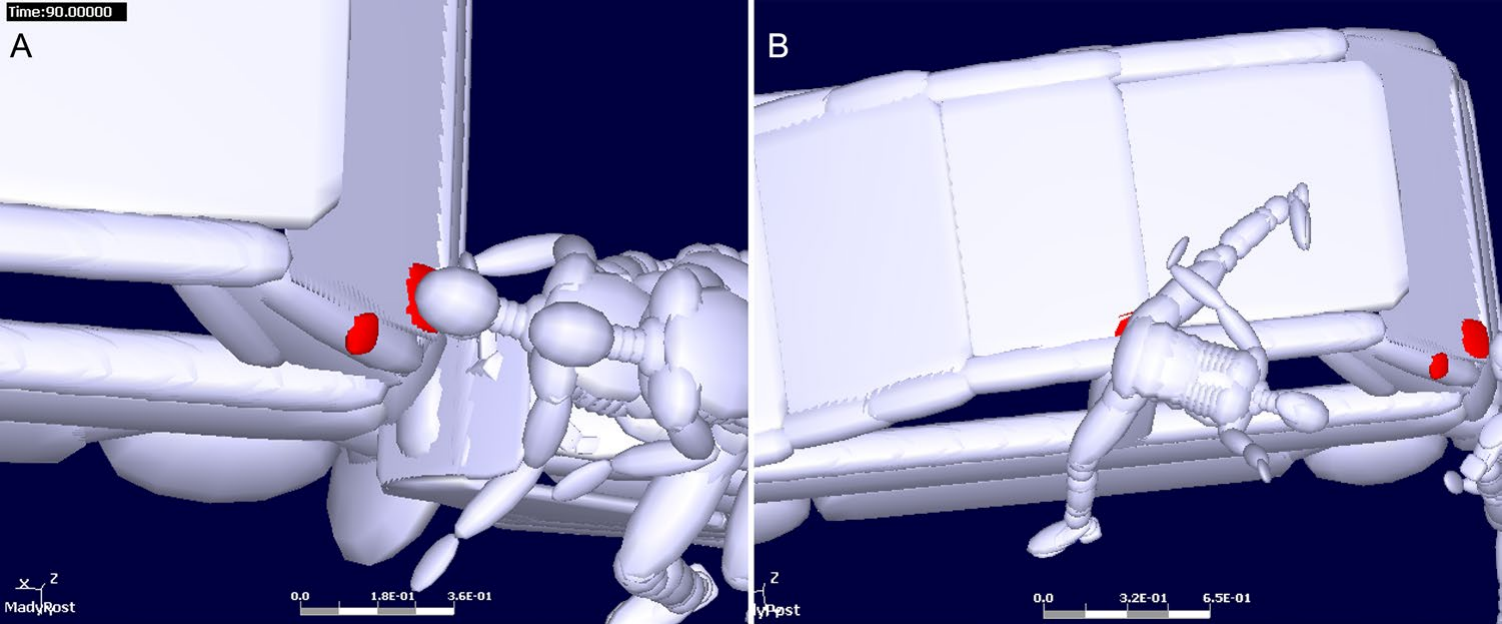
Kinematics of the motorcycle rider and passenger during collision: (A) the instant of driver contact with the minibus; (B) the instant of passenger contact with the minibus. Adapted with permission from [83].

Case 3 was a motorcycle–vehicle rear-end collision. Simulated MADYMO kinematics correlated well with accident data in this case (Figure 3); the impact positions and injury parameters correlated well with the actual injuries.

### Application in studying the mechanisms of coup and contre coup head injuries and annular fractures of the base of the skull

#### Overview

Traumatic brain injuries (TBIs) are a major cause of death in forensic cases [84]. The mechanisms of skull fractures and the patterns of brain injuries are challenging problems in forensic practice. Medical expert opinion based on experience plays a critical role in TBI cases; however, sometimes, because opinions are subjective, and because the mechanisms of head injuries are not well understood, the resulting opinions can be controversial. The FE approach is a means of attempting to better understand mechanisms of head injuries. Two examples are given herein.

#### Case example 4

**Figure 4.**
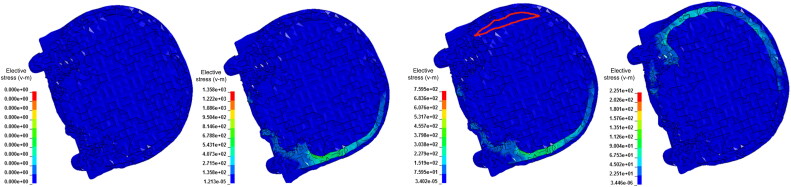
Finite element simulation of a hedge injury caused by the head’s collision with the ground. Peak strain in brain tissue at the hedge site was twice of that at the impact site.

A 50-year-old man died while fighting with others. The police were confused about whether the head injuries had been caused by a punch or by impact with the ground. AFS used an FE model of the head to simulate scenarios involving punches and ground impacts. The simulation results (Figure 4) showed that the brain injuries had most likely been caused by impact with the ground.

#### Case example 5

**Figure 5.**
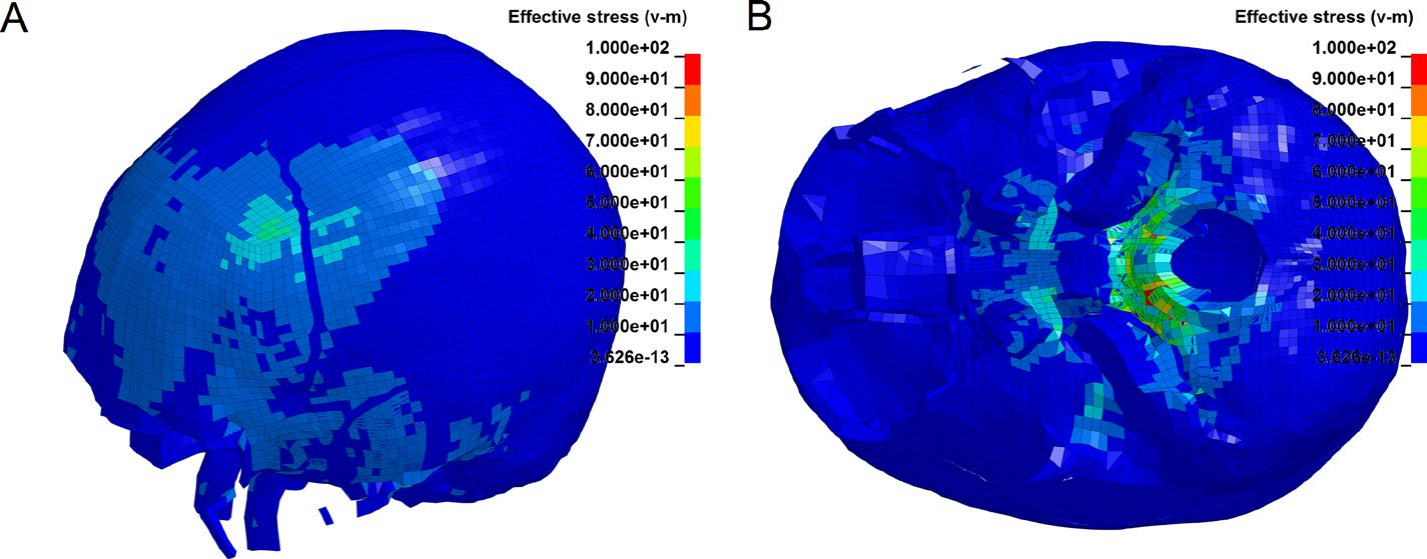
Effective stress contours of the skull (helmeted fall). (A) The effective stress increased in the left frontotemporal region of the skull. (B) The effective stress around the foramen magnum increased significantly. Adapted with permission from [78].

A woman riding a motorcycle collided with a pedestrian and fell to the ground. Several minutes later, another vehicle hit the motorcyclist. The autopsy results showed that the fatal injury was a basilar ring fracture around the foramen magnum, brain stem and cervical spinal contusions, and diffuse subarachnoid haemorrhages. The key question was to identify whether the fatal ring fracture had been caused by the initial fall or the subsequent vehicle impact. In this case [78], THUMS was used to simulate several scenarios, such as vehicle impact, falling to the ground with a helmet, and falling to the ground without a helmet. The simulation results showed that the ring fractures were most likely caused by the fall to the ground while wearing a helmet (Figure 5). The FE simulation results were consistent with the results of forensic pathology autopsy studies. Thus, it was inferred that the subsequent vehicle impact contri­buted little to the motorcyclist’s death.

### Study of the mechanisms of pelvic and lower extremity injury

#### Overview

**Figure 6.**
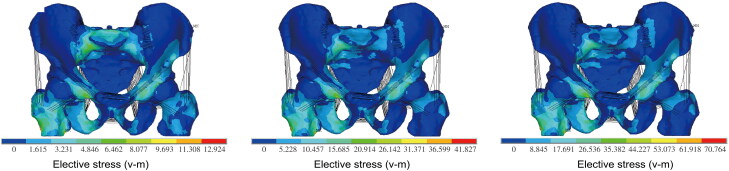
Pelvis stress distribution under different side impact loads. Adapted with permission from [85].

The lower extremity is a common site of injury in traffic accidents. Exploring the primary mechanisms of pelvis, femur, and lower extremity injury can improve the quality and accuracy of forensic investigation. AFS developed an FE model of the pelvis that included bilateral iliac bones, the sacrum, bilate­ral femurs, joint cartilage, and ligaments using DICOM format computed tomography (CT) data. Loads were applied to the trochanteric surface of the right femur to simulate a side impact [85]. The result showed that stress concentration occurred at the pubic rami (superior and inferior), hip joint, and sacroiliac joint, bilaterally (Figure 6). The model was successfully established and could be used to predict injury and provide medicolegal evidence. A case is presented to show the significance of exploring lower extremity injury mechanisms.

#### Case example 6 [86]

**Figure 7.**
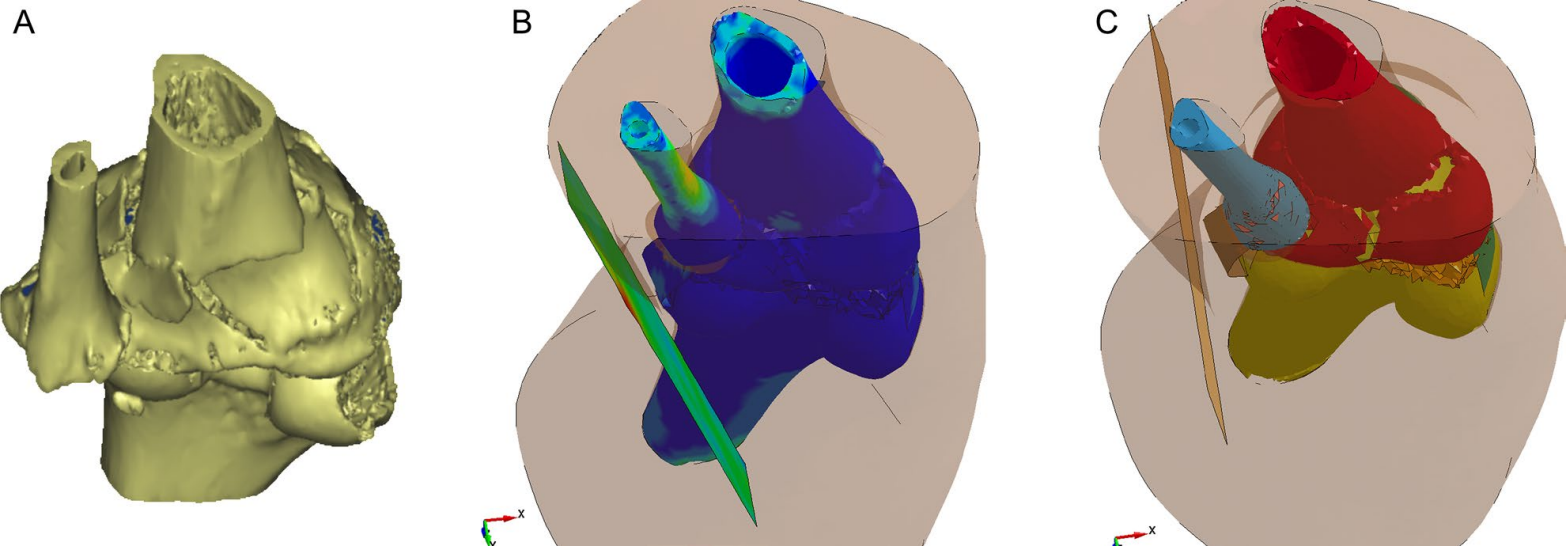
Comparison of simulation results of comminuted fracture of tibial plateau and injury caused by impact. In the case of posterolateral and lateral impingement, injury of tibial plateau and fibula (B & C) microcephaly were consistent with the injury site on CT image (A).

A woman had accused a driver of hitting and injuring her leg with his car, but the driver said that the injury had been caused from a fall to the ground when the woman climbed over the railing. AFS used an FE model of the lower limb to simulate scenarios involving the lower limbs being struck by the bumper of a car and different falls to the ground. The results showed that the injury was more likely to have been caused by direct impact, with an effect directed posterolaterally and laterally on the tibial plateau of the lower leg (Figure 7); however, the possibility of an aggravated fracture caused by falling could not be ruled out.

To determine ankle injury mechanisms in falls and traffic accidents, AFS utilised THUMS and FE to simulate falls from different heights and impacts from different directions. The results showed that falling causes a specific compression fracture of the distal tibia, whereas diaphyseal fractures of the tibia and fibula and ligament injuries caused by falling from lower heights or ankle inversion are not distinguishable from similar injury patterns caused by impact to the mid or upper leg. No obvious compression fracture of the distal tibia was caused by impact, whereas ligament injuries and avulsion fractures of the medial or lateral femoral condyles and diaphyseal fractures of the tibia and fibula were generated. Systematic studies would be helpful to analyse ankle injury circumstances and mechanisms encountered in forensic practice.

### Study of mechanical properties of cranial bone

Identifying whether a head injury was the consequence of a fall or a blow was a common question in forensic practice. The material properties of bones and brain, such as ultimate stress, ultimate strain, and elastic modulus, have a critical influence in the accuracy of computational simulations. However, there is a lack of research available on the properties of human body materials owing to the particularities of human tissue. Wang *et al.* [87] examined the differences in tissue material properties in human infants. There were no significant differences in ultimate stress, elastic modulus, or ultimate strain between the sagittal and coronal sutures, and there were significant differences in ultimate stress, elastic modulus, and ultimate strain between the frontal and parietal bones as well as between the cranial bones and sutures. How human FE model material properties can be individualised to achieve accurate simulation results is worth studying in the future.

### Crash reconstruction based on 3D image techniques and numerical simulations

#### Overview

Recently, numerical simulations are becoming a popu­lar modeling method for real-world crash reconstruction and injury evaluation. However, the use of the multi-rigid-body dynamic method depends on accurate accident data, including impact position, braking distance, driving speed, etc. The more detailed the data are, the more realistic the simulation results will be. Besides, a limited number of simulations typically involves subjective evaluations of pre-impact parameters. AFS used a multi-mode image system to obtain accurate data of accident and a real case was analysed to verify the feasibility and effectiveness of the system [88].

#### Case example 7 [88]

**Figure 8.**
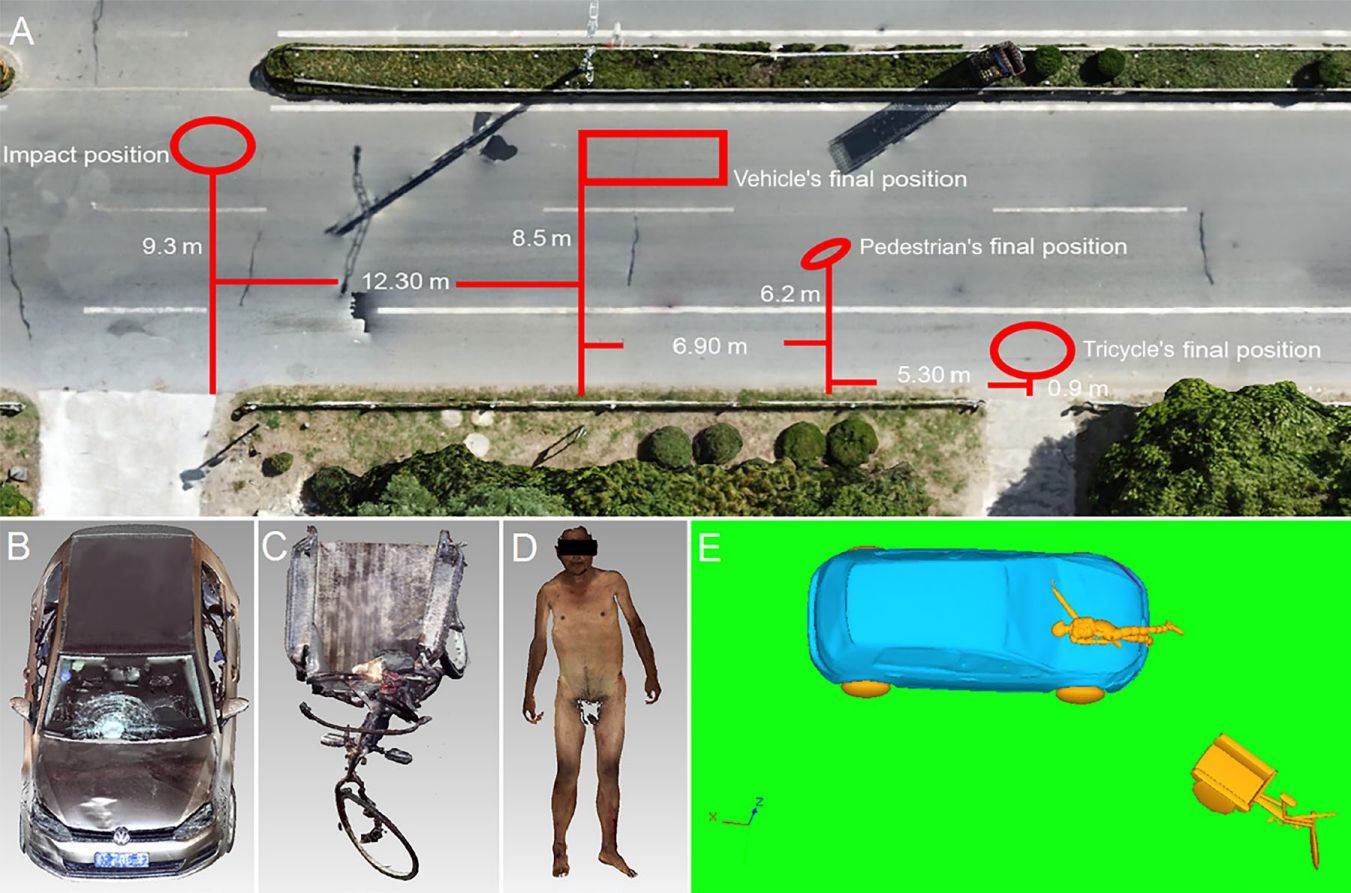
Three-dimensional documentation of the incident scene (A), vehicle (B), tricycle (C) and body surface of the victim (D), crash reconstruction result at the time of head–windshield impact (E).

This case can be summarised as follows: A pedestrian was struck by vehicle when he was pushing his tricycle across the road. AFS combined unmanned aerial vehicle (UAV) photogrammetry, laser scanning and structured-light scanning to generate 3D models of the pedestrian, vehicles, and the scene. The 3D docu­mentation of the vehicle body and incident scene saves permanent and raw material for the crash (Figure 8). Furthermore, it is an effective way to provide accurate measurements and to verify different hypotheses, for instance, corresponding deformations of vehicle to injuries. A detailed facet type multi-body of the vehicle was developed in MADYMO based on the accurate measurement by laser scanning. The simu­lation results were consistent with location of injury and vehicle. The use of 3D image techniques improves the efficiency and accuracy of numerical simulations.

### Analysis of the morphomechanical mechanism of blood dispersal based on homicide scene scanning and reconstruction

#### Overview

Blood dispersal pattern analysis is a critical component of crime scene reconstruction. Blood drops are spherical when they travel through the air. When they strike a flat surface whose angle is less than 90° (i.e. not vertical), elliptical bloodstains are formed. The morphology of the blood dispersal pattern can indicate impact angle and directionality of the blood drops, which can be used to determine the approximate location of the source. In the following case, 3D blood dispersal pattern analysis based on 3D scanning was performed.

#### Case example 8

**Figure 9.**
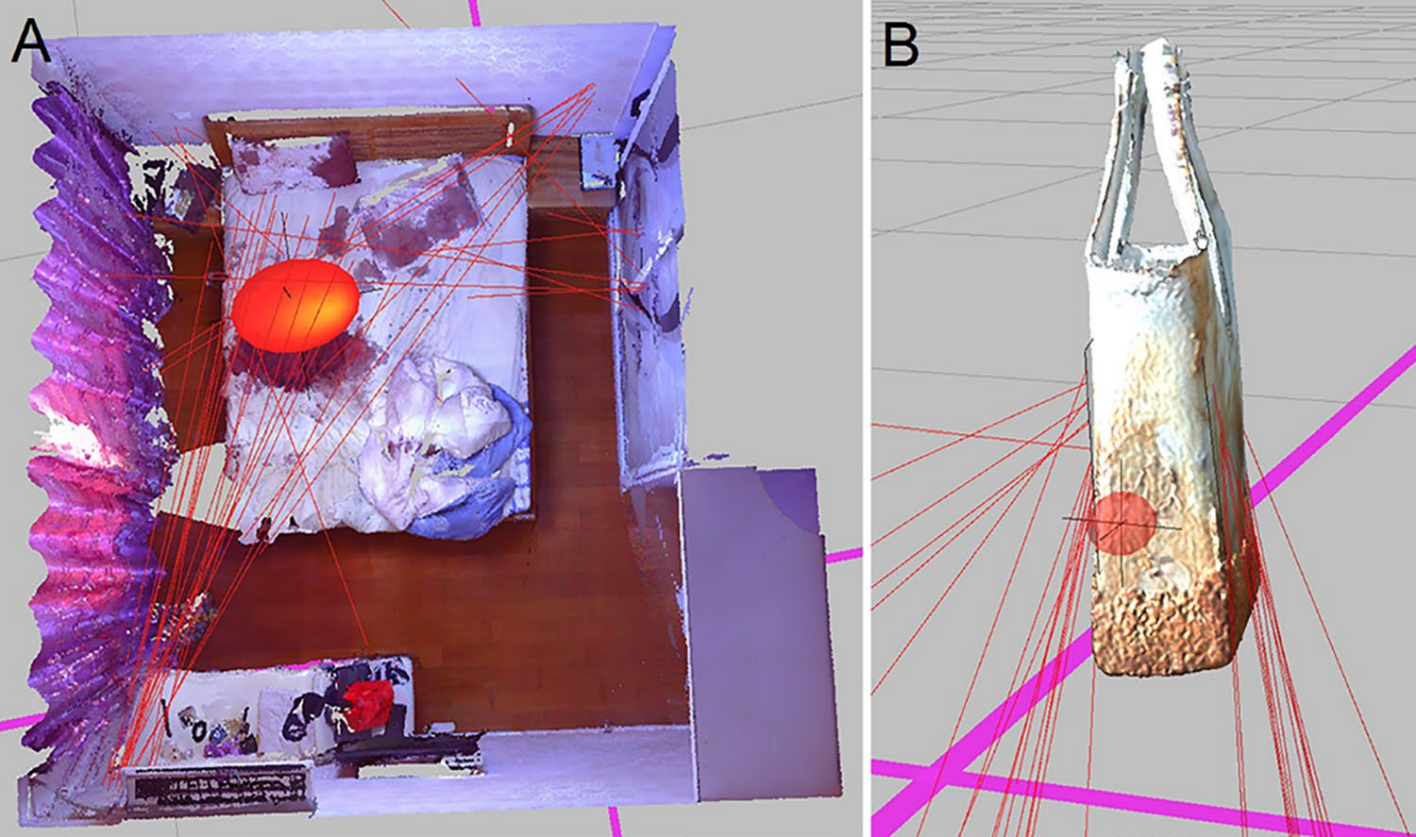
Source of blood drops in the bedroom and on the glass vase according to the bloodstain pattern analysis (BPA). (A) The sphere shows the analyzed center of the origin located around the bed where incident occurred. (B) The analyzed centre of bloodstains on the glass vase, which indicate the location of the impact. Adapted with permission from [77].

A 30-year-old woman was found lying unresponsive on the bed in the bedroom of an apartment [77]. Bloodstains were found on the walls surrounding the bed and on the bedside table, closet door, quilt, and mattress. Blood dispersal pattern analysis was conducted after a 3D reconstruction of the scene of the incident. The software uses image recognition techniques to approximate the elliptical outlines when the operator clicks on the bloodstain. The results of the analyses showed that the centre of the blood’s origin was located near the bed (Figure 9). Bloodstains were also located on the face of the glass vase, and the presence of blood provided information on the direction of the dispersal. An analysis of the bloodstain on the glass vase indicated that the centre was close to one side of the vase, which suggested that this face may have been the location of impact. This result was confirmed by the confession of the perpetrator.

## Discussion

Human body injury is common and an important part of forensic investigation and research, and the elucidation of the biomechanisms of injury is the key evidence in case characterisation for the division of responsibility, insurance claims, and case processing. Traditional forensic science mainly relied upon evidence such as characteristic injuries, field marks, and collision patterns to analyse injury mechanisms, but the injury mechanisms of complex injuries are generally difficult to determine. Based on research progress at home and abroad, forensic biomechanics has gradually overcome the limitations of empirical judgment, cadaver experiments, and animal experiments to a certain extent, and has the features of low cost, high efficiency, repeatability of different injury-causing conditions, rapid reconstruction ability of corresponding biomechanical responses, and therefore has a great advantage for the evaluation of injury mechanisms.

However, owing to the uniqueness of the human anatomy, the complexity of biological materials, and the uncertainty of injury-causing circumstances, the direct application of biomechanical research to forensic practice is still accompanied by much uncertainty:

- Human tissues have properties such as aniso­tropy, viscoelasticity, and strain rate sensitivity, while the current biomechanical models have simplified mathematical constitutive and material properties, resulting in potential discrepancies between the calculated results and the actual mechanical response. Therefore, material property data from human tissue testing, especially from materials with high strain rates, are required. In addition, the failure criteria for different organs and tissues, which are important for the evaluation of injury risk, are not sufficiently developed and require improvement.
- Current validation of biomechanical models is insufficient. Current validation of the human biomechanical models is based on a series of classical cadaveric experimental data; however in forensic practice, the varia­bility of injury-causing conditions is higher, the consequences of injury are more complex, and the validation of the existing mo­dels cannot completely cover the injuries addressed in forensic biomechanics. Therefore, we should validate forensic biomechanical models at multiple levels such as local, subsystem, and global scales; reconstruct forensic cases with clearly defined processes and conditions; further verify the validity of models using real-world data; and establish quantitative verification standards for simulation results and actual results to promote the continuous improvement of forensic biomechanical models, and thereby more accurately predict injury response and analyse injury mechanisms.
- The current accuracy of forensic biomechanics still relies upon the partial judgment of experts and the screening of loading conditions, which has good accuracy in explaining how an injury is caused but may produce false results in distinguishing the causal relationship between different injury-causing conditions, and actual injury for injury risks [4]. Therefore, in forensic biomechanics, large amounts of case reconstruction and application data are needed to carry out systematic and quantitative studies of different injury mechanisms, and observational epidemiological investigations of injury mecha­nisms need to be conducted to reduce errors in forensic biomechanics and promote forensic science applications.

Major research institutions around the world have invested a great deal of effort in forensic biomechanics research, and relying upon advances in biomechanics, the methods and models of forensic biomechanics have been improved. For Chinese research institutions, tremendous progress in forensic biomechanics modelling and practical applications have been achieved, making positive contributions to improve the probative and evidentiary value of forensic biomechanics.
